# Supplementary material for: PIAS Factors from Rainbow Trout Control NF-κB- and STAT-Dependent Gene Expression
Source: Int J Mol Sci. 2021 Nov 26;22(23):12815. doi: 10.3390/ijms222312815 (PMC8657546; doi:10.3390/ijms222312815)
Supplement: Supplementary file 1 [file ijms-22-12815-s001.zip › Figure S3.pptx]

## Slide 1
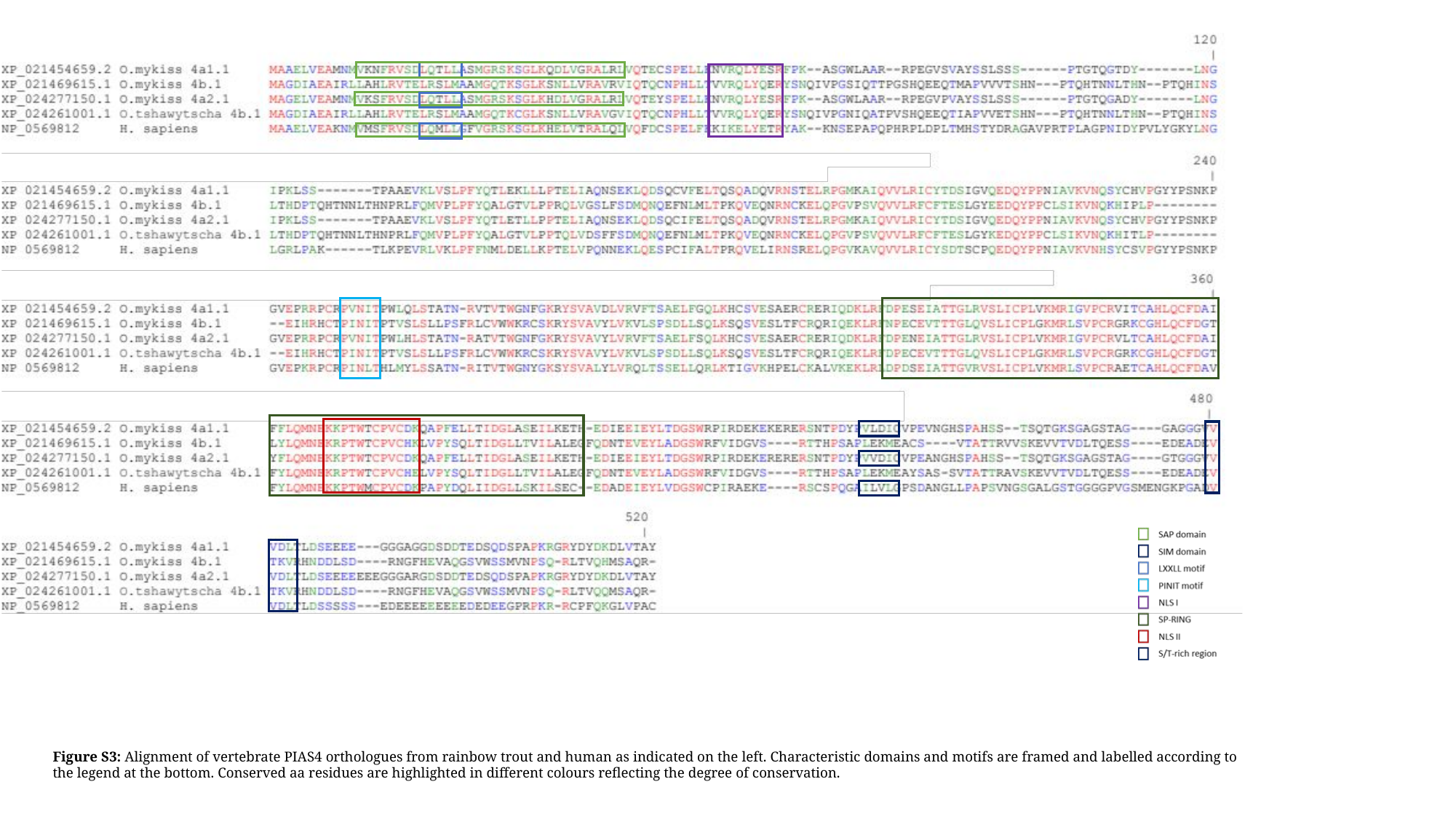

Figure S3: Alignment of vertebrate PIAS4 orthologues from rainbow trout and human as indicated on the left. Characteristic domains and motifs are framed and labelled according to the legend at the bottom. Conserved aa residues are highlighted in different colours reflecting the degree of conservation.
