# Supplementary material for: PIAS Factors from Rainbow Trout Control NF-κB- and STAT-Dependent Gene Expression
Source: Int J Mol Sci. 2021 Nov 26;22(23):12815. doi: 10.3390/ijms222312815 (PMC8657546; doi:10.3390/ijms222312815)
Supplement: Supplementary file 1 [file ijms-22-12815-s001.zip › Figure S4.pptx]

## Slide 1
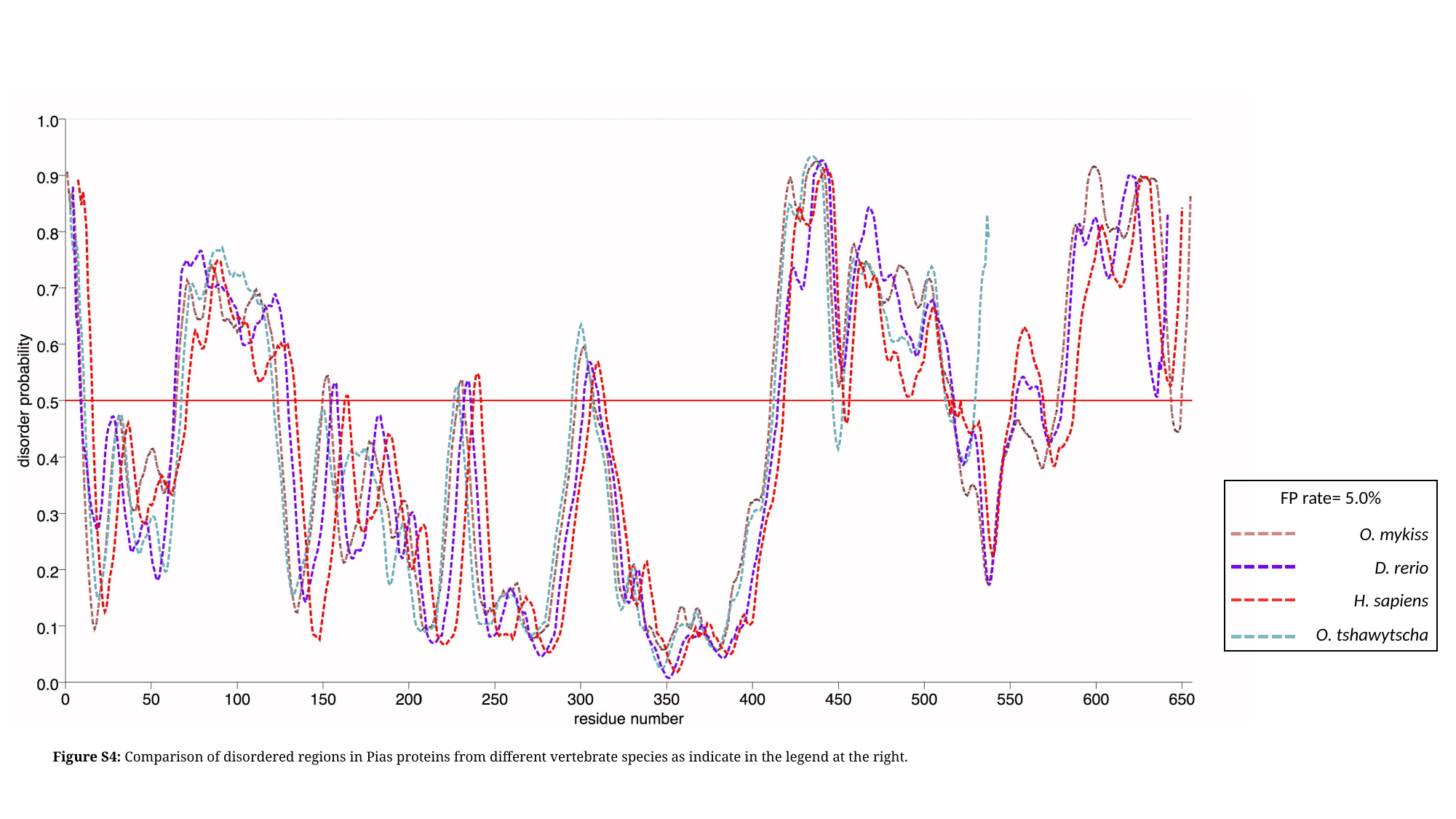

FP rate= 5.0%
O. mykiss
D. rerio
H. sapiens
O. tshawytscha
Figure S4: Comparison of disordered regions in Pias proteins from different vertebrate species as indicate in the legend at the right.
