# Supplementary material for: PIAS Factors from Rainbow Trout Control NF-κB- and STAT-Dependent Gene Expression
Source: Int J Mol Sci. 2021 Nov 26;22(23):12815. doi: 10.3390/ijms222312815 (PMC8657546; doi:10.3390/ijms222312815)
Supplement: Supplementary file 1 [file ijms-22-12815-s001.zip › Table S1.pdf]

Table S1: Cytokine-induced activation of STAT factors.

Subfamily	Teleost Cytokine	Cognate Receptor(s)	Function	Activated STATs
		Fish Type II cytokines biological interactions		
**IL-2 subfamily**	IL-2<br>IL-4/13<br>IL-7<br>IL-15<br>IL-21	IL-2Rβ1/2, IL-2/15Ra<br>IL-4Ra1/2, IL-13Ra1a/ 1b<br>IL-7Ra<br>IL-2/15Ra<br>IL-21Ra1<br>IL-21Ra1	Differentiation and homeostasis of T-cells	STAT5<br>STAT3
**β-chain cytokine subfami ly**	IL-3<br>IL-5<br>GM-CSF	IL-3Rβ/ IL-3Rα<br>IL-3Rβ/ IL-3Rα<br>GM-CSFR	Unknown in fish	STAT5<br>STAT3
**IL-6 sub-family**	IL-6<br>IL-11<br>CNTF-like<br>M17	IL-6R	Hematopoiesis	STAT3
**IL-12 subfamily**	IL-12<br>IL-23<br>IL-27<br>IL-35	IL-12 R beta 1<br>IL-12 R beta 2<br>IL-23 R<br>IL-27 R alpha<br>WSX-1<br>TCCR<br>gp130	Chemotaxis and induction of TNF-α expression	STAT4<br>STAT3/4<br>STAT1/3<br>STAT1/4
**Colony-stimulating factors**	M-CSF	CSF-1R	Inflammation	STAT1<br>STAT3<br>STAT5<br>STAT6
		Fish β-trefoil cytokines		
	IL-1β	IL-1R1, IL-1R2, DIGIRR	Inflammatory response, chemokine production	STAT3
	IL-18	IL-18R1 and IL-18R2	IFN-γ production, Th1 immunity	STAT3
	nIL-1Fm	unknown	Unknown	unknown
		Type I α-helical cytokine		
	IL-10,<br>Viral IL-10	IL-10R1/CRFB12,<br>IL-10R2/CRFB4	Inflammatory response, proliferation of T and B cells	STAT3
	IL-20L	IL-20R	Keratinocyte proliferation	STAT3
	IL-22	IL-22R1	Antibacterial and antimicrobial immunity	STAT1,<br>STAT3,<br>STAT5
	IL-26	IL-26R	Antibacterial and antimicrobial immunity	STAT1,<br>STAT3,
	IFN-a, d, e	IFNAR1/CRFB5,<br>IFNAR2/CRFB2	Apoptosis, inflammatory responds	STAT1,<br>STAT3<br>STAT3
	IFN-γ	IFN-γR1/CRFB13<br>IFN-γR2/CRFB6	Phagocyte activation,<br>Th1 cytokine expression	STAT1
	IFN-γrel	CRFB17,<br>IFN-γR2/CRFB6	Regulation of antiviral and anti-bacterial immunity	STAT1
		B-jellyroll cytokines		
	TNF-α1	TNFR2, TNFR1	Phagocytic activity of leucocytes	STAT3
	TNF-α2	TNFR2, TNFR1	T cell-mediated immunity	STAT3
	TNF-N	Unknown	Unknown	Unknown
