# Supplementary material for: PIAS Factors from Rainbow Trout Control NF-κB- and STAT-Dependent Gene Expression
Source: Int J Mol Sci. 2021 Nov 26;22(23):12815. doi: 10.3390/ijms222312815 (PMC8657546; doi:10.3390/ijms222312815)
Supplement: Supplementary file 1 [file ijms-22-12815-s001.zip › Table S2.pdf]

Table S2: Regulatory activities of the human PIAS proteins.

PIAS Family Member	Interaction Partner	Involved Domain/Motif	Function
PIAS1	AR	1-318	Sumoylates AR, co-activator or repressor
	ER-alpha	ND	Transcriptional activator
	MR	ND	Sumoylates MR, transcriptional repression
	PPAR-y	ND	Sumoylation, transcriptional repression
	SF1	ND	Sumoylation, inhibits transcriptional synergy
	GATA4	ND	Sumoylation
	SP3	ND	Sumoylation, transcriptional repression
	DNMT3A	ND	Sumoylation, releases transcriptional repression
	COUP-TFI	406-503	Co-activator
	NID	ND	Sumoylation, transcriptional repression
	ZNF76	1-277	Sumoylation, releases inhibition of ZNF76 on TBP
	BKLF	ND	Sumoylation, transcriptional repression
	AXIN	ND	Sumoylation, JNK activation
	Dynamin	ND	Sumoylation, endocytosis
	FAK	ND	Sumoylation, activates autophosphorylation of FAK
	SNM1A	ND	Proper nuclear localization and DNA repair
	CRP2	ND	ND
PIAS2	AR	RLD	Sumoylates AR, activator or repressor
	GR	RLD	Transcriptional repression
	PPAR-y	ND	Sumoylation, transcriptional repression
	GRIP1	RLD	Transcriptional co-activator
	DJ1	433-493	DJ1 releases PIASx-α-mediated repression on AR
	SF1	ND	Sumoylation, regulates nuclear localization
	HDAC3	ND	Relieves transcriptional repression by HDAC3
	DNMT3A	ND	Sumoylation, releases transcriptional repression
	BKLF	ND	Sumoylation, transcriptional repression
	AXIN	ND	Sumoylation, JNK activation
PIAS3	AR	ND	Transcriptional repression
	MR	444–583	Transcriptional repression
	TIF2	353–546	Modulates TIF2 activity in NR signaling
	SF1	ND	Sumoylation, inhibits transcriptional synergy
	p300	ND	Activates SMAD3-mediated transcription
	HMGI-C	ND	Transcriptional repression
PIAS4	AR	336-406	AR corepressor
	PR	ND	Transcriptional repression
	NURR1	ND	Sumoylation, transcriptional repression
	SF1	ND	Sumoylation, regulates nuclear localization
	HDAC1, -2	1-90	Represses SMAD3- and AR-mediated transcription
	GATA1	ND	Sumoylation, transcriptional repression
	GATA2	1–157, 414–510	Sumoylation, transcriptional repression
	C/EBP-α	ND	Sumoylation, inhibits transcriptional synergy
	TCF4	ND	Sumoylation, transcriptional activation
	TRAF6	SAP/PINIT	Sumoylation, transcriptional regulation

Abbreviations: AR, androgen receptor; AXIN, axis inhibitor; BKLF, basic Krüppel-like factor; CBP, cyclic-AMP-responsive-element-binding protein (CREB)-binding protein; C/EBP-α, CCAAT/enhancer binding protein-α; COUP-TFI, chicken ovalbumin upstream-promoter-transcription factor 1; CRP2, cysteine-rich protein 2; DNMT3A, DNA methyltransferase 3A; ER-α, oestrogen receptor-α; FAK, focal adhesion kinase; GATA, GATA-binding protein; GBP, GU/RH-II-binding protein; GR, glucocorticoid receptor; GRIP1, glutamate-receptor-interacting protein 1; GU/RH-II, nucleolar RNA helicase GU/RNA helicase II; HDAC, histone deacetylase; HMGI-C, high-mobility group protein isoform I-C; JNK, JUN amino-terminal kinase; MIZ1, MSX-interacting zinc finger 1; MR, mineralocorticoid receptor; MSX2, MSH-homeobox homologue 2; ND, not determined; NID, net inhibitory domain; NR, nuclear receptor; NURR1, nuclear-receptor-related protein 1; PIAS, protein inhibitor of activated STAT (signal transducer and activator of transcription protein); PPAR-γ, peroxisome-proliferative-activated receptor-γ; PR, progesterone receptor; RLD, RING-finger-like zingbinding domain; SF1, steroidogenic factor 1; SMAD, SMA (small body size)- and MAD (mothers against decapentaplegic)- related protein; TBP, TATA-binding protein; TCF4, T-cell-specific transcription factor 4; TIF2, transcriptional intermediary factor 2; ZNF76, zinc-finger protein 76.
